# Reduced virulence of the MARTX toxin increases the persistence of outbreak-associated *Vibrio vulnificus* in host reservoirs

**DOI:** 10.1016/j.jbc.2021.100777

**Published:** 2021-05-14

**Authors:** Sanghyeon Choi, Byoung Sik Kim, Jungwon Hwang, Myung Hee Kim

**Affiliations:** 1 Infection and Immunity Research Laboratory, Metabolic Regulation Research Center, Korea Research Institute of Bioscience and Biotechnology (KRIBB), Daejeon, Korea; 2 Department of Biological Sciences, Korea Advanced Institute of Science and Technology, Daejeon, Korea; 3 Department of Food Science and Engineering, Ewha Womans University, Seoul, Korea

**Keywords:** MARTX toxin, effector, cysteine protease domain, *Vibrio vulnificus*, virulence factor, infectious disease, ABH, the alpha/beta hydrolase domain, ARF, ADP-ribosylation factor, CD, circular dichroism, CPD, cysteine protease domain, DmX, domain X effector, DUF1, domain of unknown function, ExoY, ExoY-like adenylate cyclase domain, hpi, hours postinfection, MARTX, multifunctional autoprocessing repeats-in-toxin, MCF, makes caterpillars floppy-like, RID, Rho GTPase-inactivation domain

## Abstract

Opportunistic bacteria strategically dampen their virulence to allow them to survive and propagate in hosts. However, the molecular mechanisms underlying virulence control are not clearly understood. Here, we found that the opportunistic pathogen *Vibrio vulnificus* biotype 3, which caused an outbreak of severe wound and intestinal infections associated with farmed tilapia, secretes significantly less virulent multifunctional autoprocessing repeats-in-toxin (MARTX) toxin, which is the most critical virulence factor in other clinical *Vibrio* strains. The biotype 3 MARTX toxin contains a cysteine protease domain (CPD) evolutionarily retaining a unique autocleavage site and a distinct β-flap region. CPD autoproteolytic activity is attenuated following its autocleavage because of the β-flap region. This β-flap blocks the active site, disabling further autoproteolytic processing and release of the modularly structured effector domains within the toxin. Expression of this altered CPD consequently results in attenuated release of effectors by the toxin and significantly reduces the virulence of *V. vulnificus* biotype 3 in cells and in mice. Bioinformatic analysis revealed that this virulence mechanism is shared in all biotype 3 strains. Thus, these data provide new insights into the mechanisms by which opportunistic bacteria persist in an environmental reservoir, prolonging the potential to cause outbreaks.

Unlike obligate pathogens, opportunistic pathogens evolve to increase their fitness in environmental hosts by controlling their virulence, which may facilitate host-to-host spreading (1, 2). *Vibrio vulnificus* is a life-threatening opportunistic pathogen that is becoming an increasing threat to human health worldwide because of global climate change (3, 4). *V. vulnificus*-associated infections mostly occur through consumption of contaminated seafood and can result in primary septicemia with a high fatality rate (∼50%) in some severe cases (5, 6). Exposure of open wounds to infected sea water or seafood products, which can cause wound infections and secondary septicemia, is also associated with a substantial mortality rate (∼25%) (7). Strains of *V. vulnificus* are classified into three biotypes based on their biochemical characteristics and phylogeny: biotype 1 strains such as MO6-24/O and CMCP6 cause the majority of human infections responsible for the entire spectrum of illness, including primary septicemia; biotype 2 strains are primarily eel pathogens; and biotype 3 strains, such as strain BAA87, cause wound infections and bacteremia and possess hybrid biochemical properties of both biotypes 1 and 2 (3, 4).

Biotype 3 strains have been exclusively isolated from outbreaks of severe wound infections and septicemia cases associated with a tilapia farm in Israel (8). Comparative genomic analysis revealed that biotype 3 is a distinct clone descended from the parental environmental population that acquired pathogenic potential by horizontal gene transfer from other *Vibrio* strains (9).

Meanwhile, the biotype 3 strains are less pathogenic than the biotype 1 strains (10). Among virulence factors produced by *V. vulnificus* strains, multifunctional autoprocessing repeats-in-toxin (MARTX) toxins are the primary exotoxins that regulate host inflammatory responses and immune defense (11) and facilitate colonization in the intestine and dissemination to distal organs (12, 13, 14, 15). The biotype 3 MARTX toxin harbors a distinct effector content derived from a putative progenitor MARTX toxin of the biotype 1 strain that contributes to the significantly reduced virulence of the strain (10). This study suggests that rather than showing increased potency, outbreak strains may retain decreased potency of key virulence factors as a strategy to enter the human food chain by persisting longer in natural hosts (10). However, the mechanisms by which the distinct effector content of the biotype 3 MARTX toxin leads to reduced pathogenicity are unclear.

MARTX toxins secreted by many bacterial pathogens contain disease-related, modularly structured effector domains that are released *via* processing events upon entry into host cells (16, 17, 18). The diversity of MARTX toxin effector domains correlates with distinct cytopathicities or cytotoxicities and with the overall toxicity of MARTX toxin-expressing strains (19, 20). The cysteine protease domain (CPD) located at the end of the effector domain regions of all MARTX toxins directs the proteolytic processing of effector modules after it undergoes activation and autocleavage by binding to cytosolic inositol hexakisphosphate (InsP_6_) (17, 18). The makes caterpillars floppy-like (MCF) effector domain, which is present in about 30% of MARTX toxins, leads to effector module processing after binding to ADP-ribosylation factor (ARF) (16).

Our previous study revealed that effector domains within the biotype 3 MARTX toxin are unusually processed by its internal CPD and domain X effector (DmX, a homolog of MCF) (16). The CPD of biotype 3 MARTX toxin lacks the proteolytic function to process its associated effector domains, even though it has autocleavage activity in the presence of InsP_6_. In addition, only DmX is released from the toxin by the allosteric activator ARF after autocleavage and detachment from the autocleaved CPD, suggesting that these distinctive processing pathways may be correlated with the toxin potency and therefore the reduced virulence of this strain.

These previous data led us to hypothesize that the opportunistic biotype 3 strain has evolved to promote fitness in the host (*e.g.*, tilapia) by relieving MARTX toxin-mediated virulence. In this study, we found that the biotype 3 MARTX toxin CPD evolved to contain an atypical N-terminal autocleavage site and a distinct C-terminal β-flap region that attenuates the autoproteolytic activity of the CPD required for the processing of associated effector domains, which leads to significantly reduced virulence in cells and mice. Bioinformatics analysis suggests that the *V. vulnificus* biotype 3 strains evolved to retain the atypical CPD and thus have lower MARTX toxin–driven pathogenicity than biotype 1 and 2 strains, which maintain the functional MARTX toxin and exhibit potent virulence.

## Results

### The biotype 3 MARTX toxin CPD is autocleaved atypically

**Figure 1:**
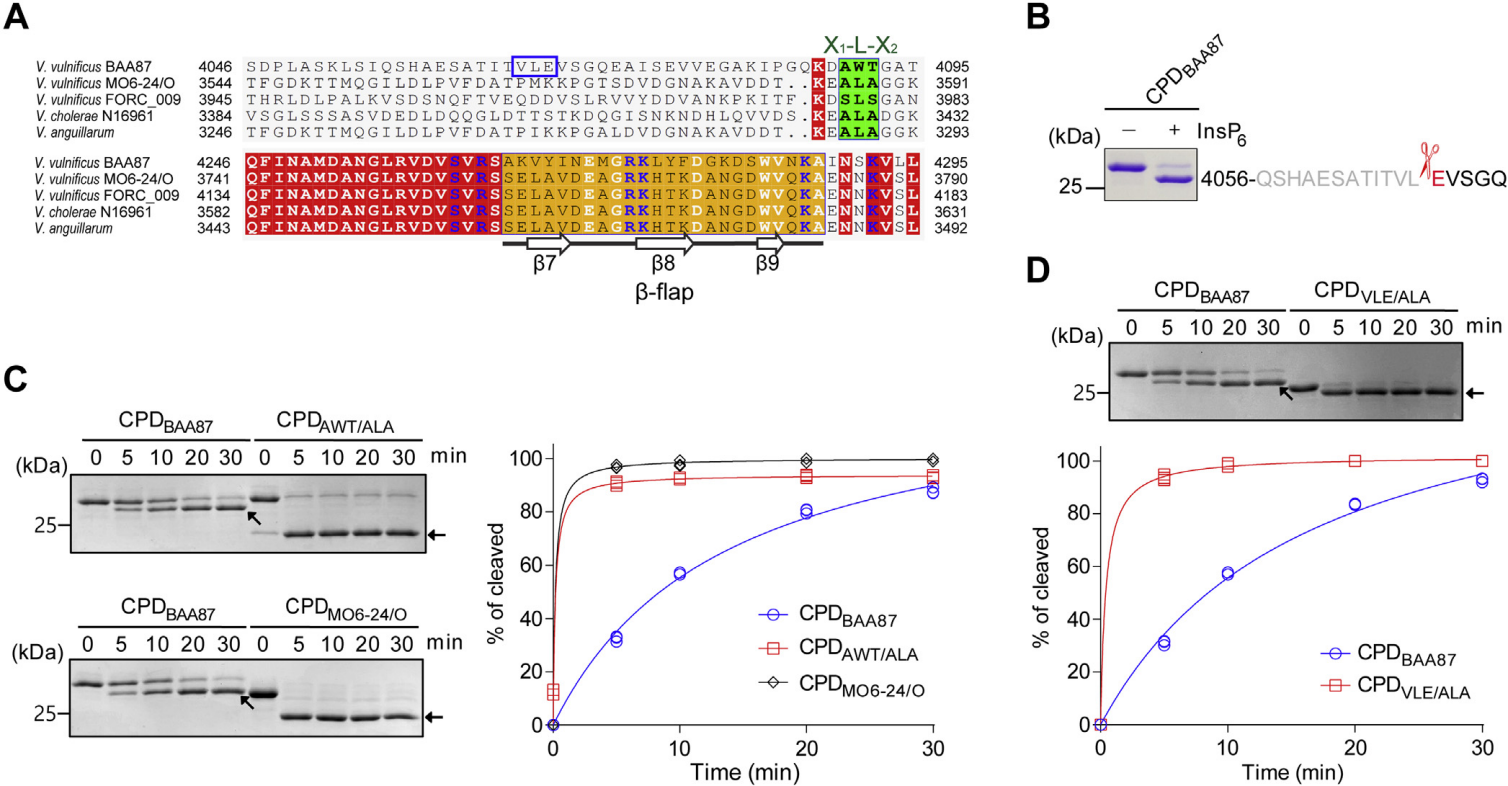
**The CPD of the *V. vulnificus* biotype 3 MARTX toxin is autocleaved atypically.***A*, multiple sequence alignment of CPDs of MARTX toxins from various *Vibrio* species. Completely conserved residues are highlighted in *red*, sequences for the β-flap region are indicated in *dark yellow*, X_1_-L-X_2_ motifs (autocleavage sites) are displayed in *green*, and autocleavage residues (VLE) of the *V. vulnificus* BAA87 CPD are indicated in *blue*. Within the β-flap region, secondary structure elements of the *V. cholerae* CPD (PDB ID 3EEB) are indicated. Multiple sequence alignment was carried out using MultiAlin, and the figure was generated with ESPript. The following *Vibrio* strains are shown: *V. vulnificus* BAA87 (WP_039507922.1), *V. vulnificus* MO6-24/O (WP_015728045.1), *V. vulnificus* FORC_009 (WP_060534095.1), *V. cholerae* N16961 (AAD21057.1), and *V. anguillarum* (RMZ64238.1). *B*, autocleavage site analysis of CPD_BAA87_. The N-terminal sequence of autocleaved CPD_BAA87_ is EVSGQ, as determined by Edman sequencing. The first amino acid (E) of the autocleaved CPD is highlighted in *red*. *C* and *D*, *in vitro* autocleavage assay. CPD_BAA87_, its mutants CPD_AWT/ALA_ and CPD_VLE/ALA_, and CPD_MO6-24/O_ were incubated with equivalent molar concentrations of InsP_6_, resolved by SDS-PAGE, and visualized by Coomassie staining. Autocleaved CPDs are indicated by *arrows*. The intensity of the cleaved protein on SDS-PAGE was quantified using NIH ImageJ and plotted. The plots are from three independent experiments. CPD, cysteine protease domain; MARTX, multifunctional autoprocessing repeats-in-toxin.

Our previous study showed that the internal CPD (CPD_BAA87_) of MARTX toxin secreted by the *V. vulnificus* biotype 3 clinical strain BAA87 (MARTX_BAA87_) does not process its associated effector domains, even though CPD_BAA87_ itself is autocleaved in the presence of InsP_6_ (16). A multiple sequence alignment of CPDs within MARTX toxins expressed by different *Vibrio* species, including *V. vulnificus* and *Vibrio cholerae*, revealed that CPD_BAA87_ has a distinct unique sequence (A_4090_-W_4091_-T_4092_) instead of the conventional cleavage sequence, which is known as an X_1_-L-X_2_ motif (where X_1_ and X_2_ are small amino acids such as Ala and Ser) (21) (Fig. 1*A*). The other distinctive feature of CPD_BAA87_ is the C-terminal region corresponding to the β-flap in the CPD (CPD_cholerae_) of the *V. cholerae* MARTX toxin, which plays a critical role in InsP_6_ binding and enzymatic function (17). The amino acid sequence of this region in CPD_BAA87_ is significantly different from that of other CPDs (Fig. 1*A*).

To identify the autocleavage site of CPD_BAA87_, we carried out an *in vitro* autocleavage assay using N-terminally extended CPD_BAA87_ in the presence of InsP_6_ and analyzed the product by Edman sequencing. As expected, CPD_BAA87_ was not processed at the unique sequence AWT. Rather, it was autocleaved between Leu4067 and Glu4068 (Fig. 1*B*), which is located 24 amino acids upstream of X_1_-L-X_2_, the conventional autocleavage site of other CPDs (Fig. 1*A*). Based on these results, we hypothesized that CPD_BAA87_ may have evolved to function differently from other CPDs to control the virulence of the biotype 3 strain *via* the MARTX toxin.

To test this hypothesis, we mutated the unique sequence AWT to ALA and analyzed the autocleavage activity. The mutant CPD (CPD_AWT/ALA_) was preferentially and efficiently (comparable activity with CPD_MO6-24/O_ of the MO6-24/O strain) autocleaved at the ALA site rather than at its authentic cleavage site, VLE (Fig. 1*C*). The cleavage sequence, ALA, was confirmed by Edman sequencing. The autocleavage activity at the VLE site was much weaker in CPD_BAA87_ than in CPD_MO6-24/O_ (Fig. 1*C*). Noticeably, the autocleavage motif of CPD_BAA87_ contains a bulky side chain residue (glutamate), whereas the conventional cleavage motif has small amino acid residues at positions X_1_ and X_2_ (21), suggesting that this difference may cause the reduced autocleavage activity. Substitution of the VLE motif with ALA increased the autocleavage activity to a lever higher than that of WT CPD_BAA87_ (Fig. 1*D*). It should be noted that migration of the mutant CPD by sodium dodecyl sulfate (SDS)-polyacrylamide gel electrophoresis (PAGE) was faster than that of WT CPD_BAA87_. Collectively, these data suggest that CPD_BAA87_ is an altered CPD that evolved to moderate the virulence of the MARTX toxin.

### The structure of CPD_BAA87_ reveals nonfunctionality for processing of associated effectors

To uncover the molecular basis of the reduced function of CPD_BAA87_ with respect to both its autocleavage and the processing of associated effectors, we initially tried to crystallize the autocleaved CPD_BAA87_, covering Glu4068–Asp4300, but could ultimately only crystallize CPD by deletion of its N-terminal region (Ala4090–Asp4300), suggesting that the N-terminal Glu4068–Asp4089 region may be flexible and not crystallizable. Its structure was determined at a resolution of 2.2 Å (Table S1). It should be noted that the expression construct of CPD_BAA87_ for crystallization included an N-terminal hexahistidine-tag, a tobacco etch virus protease recognition site, and restriction enzyme cloning site-derived residues (AM), followed by the unique sequence A_4090_-W_4091_-T_4092_ (Fig. S1*A*). When incubated with InsP_6_, the CPD was autocleaved at the AMA_4090_ site because of its high similarity to the conventional CPD autocleavage sequence (ALA). Purified CPD starts at Ala4090 (Fig. S1*B*), indicating that the determined structure is a representative autocleaved conformation of CPD_BAA87_ lacking the flexible N-terminal Glu4068–Asp4089 fragment.

**Figure 2:**
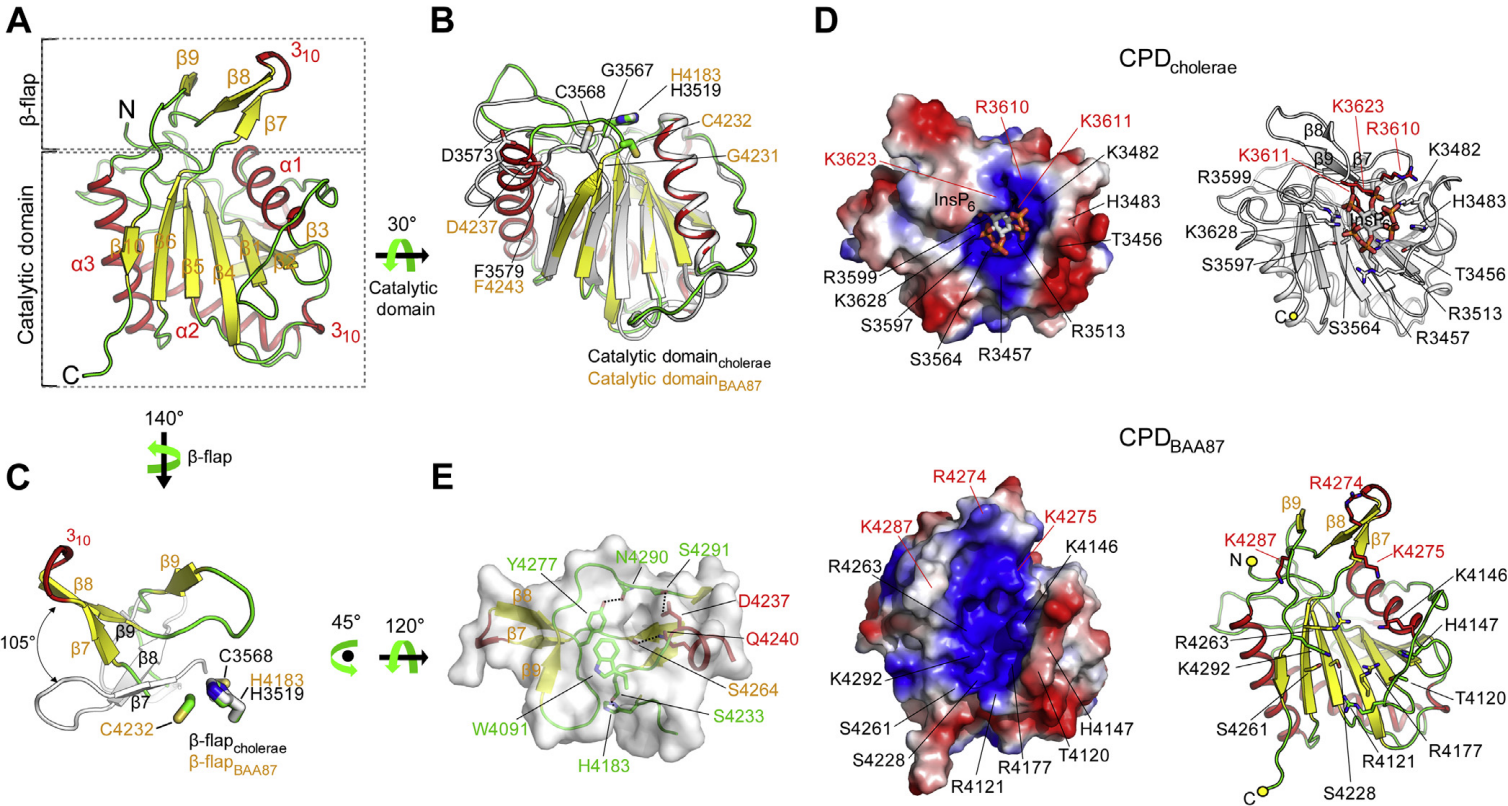
**The structure of CPD**_**BAA87**_**suggests nonfunctionality for processing of effector domains.***A*, crystal structure of CPD_BAA87_, consisting of the core catalytic domain and β-flap. The helices, β-strands, and loop regions are shown in *red*, *yellow*, and *green*, respectively. *B*, superimposition of the catalytic domain of CPD_BAA87_ onto the corresponding domain of CPD_cholerae_ (*white*). Residues corresponding to different secondary structures of CPD_BAA87_ are shown in *color*, and residues corresponding to CPD_cholerae_ are shown in *black*. The catalytic residues of CPD_BAA87_ (H4183 and C4232) and CPD_cholerae_ (H3519 and C3568) are indicated. *C*, superimposition of the β-flap of CPD_BAA87_ onto the corresponding region of CPD_cholerae_ (*white*). Active residues are indicated for each CPD. *D*, the electrostatic surface potential of CPD_cholerae_ shows a positively charged pocket for InsP_6_ binding (*upper left panel*). The residues involved in formation of the pocket are indicated. Residues involved in InsP_6_ binding are shown as a *stick model* within CPD_cholerae_ displayed as a *ribbon model* (*upper right panel*). The β-flap_cholerae_ residues critically involved in formation of the InsP_6_-binding pocket are indicated in *red* (*upper panels*). The electrostatic surface potential of CPD_BAA87_ reveals a highly basic pocket formed by the corresponding residues of CPD_cholerae_ (*lower left panel*). The corresponding residues involved in the InsP_6_-binding pocket of CPD_BAA87_ are displayed, including the residues corresponding to the critical β-flap_cholerae_ residues for InsP_6_ binding, which are shown in *red* (*lower right panel*). *E*, crater-like hydrophobic surface formed by structural movement of the β-hairpin in the β-flap region *via* interacting forces. Hydrogen bonds between residues are shown as *dashed lines*. CPD, cysteine protease domain; MARTX, multifunctional autoprocessing repeats-in-toxin.

The overall structure of the CPD_BAA87_ is similar to that of CPD_cholerae_. Its catalytic domain (Asn4099–Ser4262), which shows the most similarity with CPD_cholerae_, contains a seven-stranded β-sheet with five central parallel strands and two antiparallel capping strands flanked by three helices (Fig. 2, *A* and *B*). CPD_BAA87_ also contains a β-flap region (Ala4265–Lys4288) that has an entirely different conformation from the corresponding region of CPD_cholerae_ (Fig. 2, *A* and *C* and Fig. S2). Although InsP_6_ binds to CPD_BAA87_ covering residues 4056 to 4300 and residues 4090 to 4300 with binding affinity constants (*K*_d_) of 3.95 and 2.43 μM, respectively (Fig. S3), an electron density map of the molecule could not be found, and thus, the structure model does not include InsP_6_ (see below).

CPD_cholerae_ forms a positively charged pocket for InsP_6_ binding that is required for active site opening (17). The structural integrity of the β-flap in CPD_cholerae_ is essential for forming both the pocket and the substrate-binding cleft adjacent to the dyad active residues. The residues Arg3610, Lys3611, and Lys3623 on the β-flap play critical roles in maintaining the integrity of the β-flap (Fig. 2*D*, upper panels) (17). Although all residues involved in formation of the InsP_6_-binding pocket in CPD_cholerae_ are strictly conserved in CPD_BAA87_, including Arg3610, Lys3611, and Lys3623, the structure of CPD_BAA87_ reveals a dissimilar conformation of the pocket (Fig. 2*D*, lower panels). In the structure of CPD_cholerae_, the region Gly3567–Phe3579, which includes the active residue Cys3568, forms a flexible long loop and exposes the active site to solvent (Fig. 2*B*). CPD_BAA87_ shows a different structural conformation in that the C terminal half of the corresponding region Asp4237–Phe4243 (Asp3573–Phe3579 in CPD_cholerae_) is incorporated into the third helix of CPD_BAA87_, resulting in a longer helix (Fig. 2*B*). Furthermore, the β-hairpin in the β-flap region in CPD_BAA87_ is rotated about 105° clockwise relative to the corresponding β-hairpin in CPD_cholerae_ (Fig. 2*C*). These structural changes in CPD_BAA87_ are facilitated by several hydrogen bonds between His4183 and Ser4233, Gln4240 and Ser4264, Asp4237 and Ser4291, and Tyr4277 and Asn4290, as well as successive layers of hydrophobic interactions that allow the N-terminal Trp4091 to cover the crater-like hydrophobic surface (Fig. 2*E*). These events consequently change the locations of Arg4274, Lys4275, and Lys4287, which correspond to the residues Arg3610, Lys3611, and Lys3623 on the β-flap in CPD_cholerae_, moving them further from the InsP_6_-binding site (Fig. 2*D*, lower panels) and force the active residue Cys4232 into an improper position away from the other active residue, His4183 (Fig. 2*C*).

**Figure 3:**
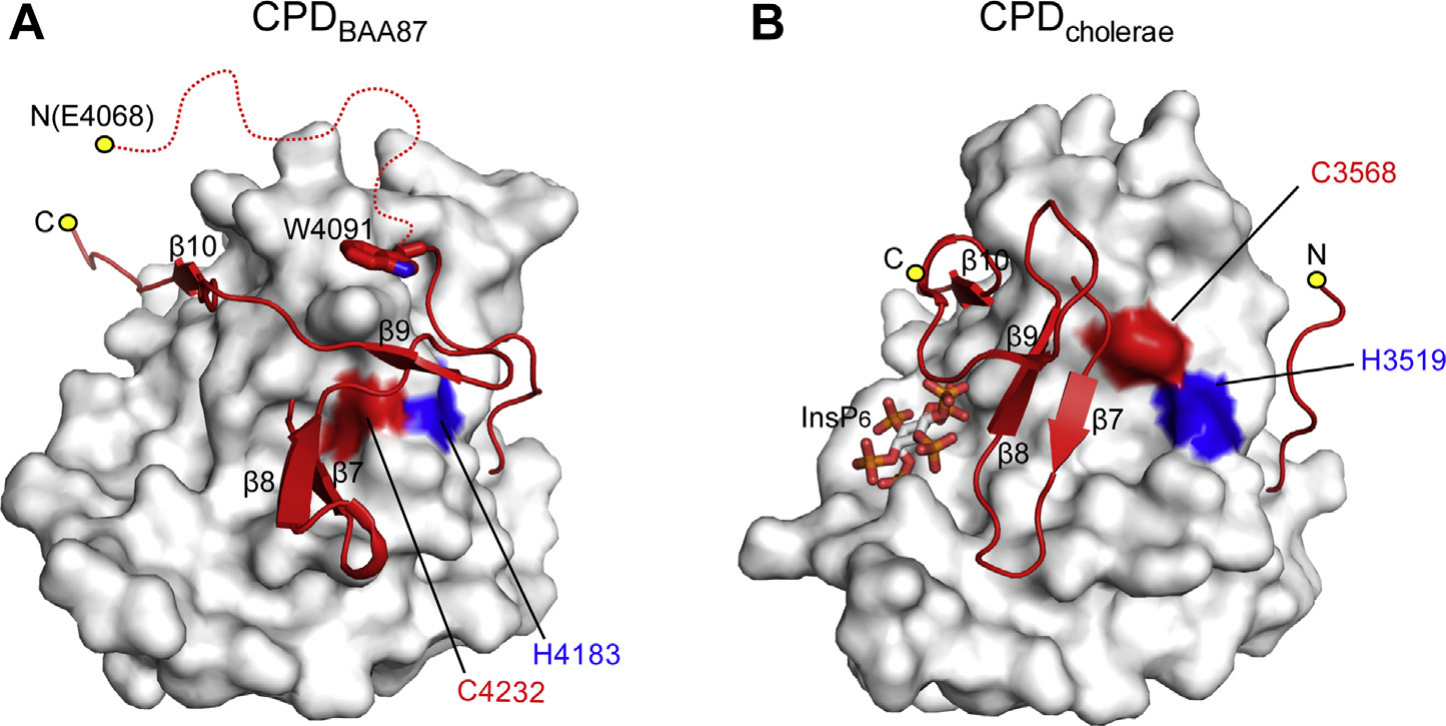
**Autocleaved CPD**_**BAA87**_**becomes nonfunctional for processing of MARTX toxin effectors.***A*, the steric configuration of the β-flap (*ribbon model* in *red*) blocking the active site of the autocleaved CPD_BAA87_. Catalytic residues are shown in *red* (Cys4232) and *blue* (His4183) on a surface model of the catalytic domain (in *white*). The N-terminal flexible region (Glu4068–Ala4090) is shown as a *red dashed line*, and Trp4091 is shown with a *red stick model*. *B*, the steric configuration of the β-flap, showing autocleaved CPD_cholerae_, which is functional for processing of MARTX toxin effectors. Catalytic residues are shown in *red* (Cys3568) and *blue* (His3519) on the surface model of the catalytic domain (in *white*). The two structures are displayed in the same orientation. CPD, cysteine protease domain; MARTX, multifunctional autoprocessing repeats-in-toxin.

Overall, these data suggest that CPD_BAA87_ that is autocleaved at the site V_4066_-L_4067_-E_4068_ constitutes an atypically structured CPD because of significant conformational changes in the N-terminal flexible region spanning Glu4068–Ala4090, the Trp4091 that caps the C-terminal hydrophobic region following the β-hairpin, the conserved catalytic core domain, and the C-terminal β-flap region, which forms a shield together with Trp4091 to block the active site (Fig. 3*A*). Consequently, unlike autocleaved CPD_cholerae_ (Fig. 3*B*), the autocleaved CPD_BAA87_ becomes nonfunctional with respect to the processing of linker regions between associated effector domains within the MARTX toxin.

We believe that the unavailability of an electron density map of InsP_6_ may be a consequence of the significantly changed conformation of the β-flap region after autocleavage of CPD_BAA87_ (Fig. 3, *A* and *B*), which alters the locations of residues critical for InsP_6_ binding (Arg4274, Lys4275, and Lys4287) (Fig. 2*D*). Mutation of Lys3611 or Lys3623 in CPD_cholerae_, which correspond to Lys4275 and Lys4287 in CPD _BAA87_, abolished InsP_6_ binding (17).

### Evolutionary insights into inhibition of MARTX toxin activation by CPD_BAA87_

**Figure 4:**
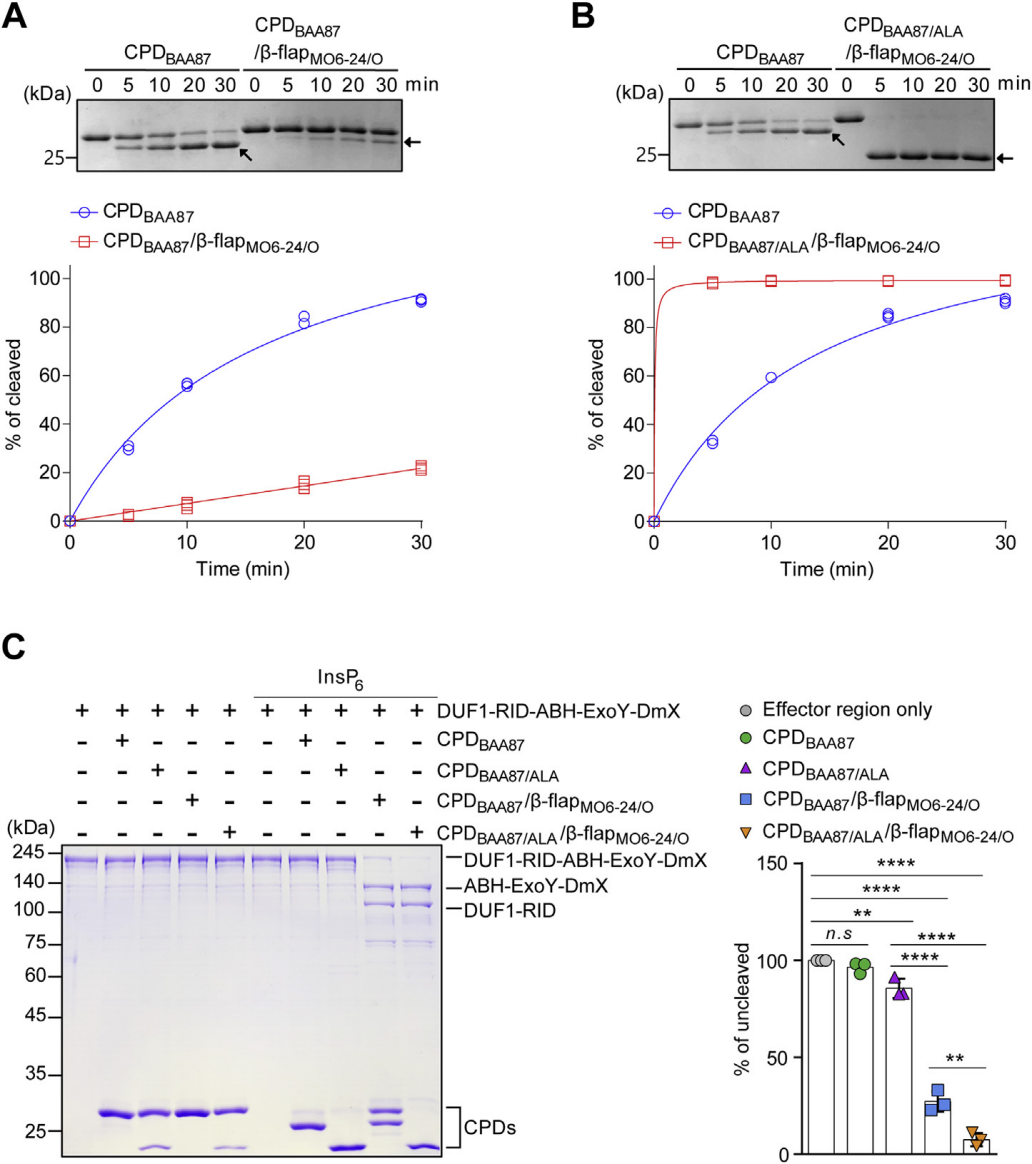
**Evolutionary insights into the disabled effector domain processing of CPD**_**BAA87**_**.***A* and *B*, *in vitro* autocleavage assay for CPD proteins. CPDs were incubated with InsP_6_ for the indicated durations. The intensities of the bands representing the cleaved protein on SDS-PAGE were quantified with NIH ImageJ and plotted. The plots are from three independent experiments. Autocleaved CPDs are indicated by *arrows*. *C*, *in vitro* MARTX effector domain processing assay. The purified effector region of MARTX_BAA87_ toxin was co-incubated with the indicated CPDs in the presence of 1 mM InsP_6_ for 1 h at 37 °C. Effector products processed by each CPD were analyzed by SDS-PAGE (*left panel*). The bar graph shows the proportion of the uncleaved effector region relative to the effector region in the absence of CPD (*right panel*). The intensity of each band representing the uncleaved effector region was quantified with NIH ImageJ, and the mean of individual values were statistically analyzed by a one-way ANOVA with multiple comparisons (∗∗*p* < 0.005; ∗∗∗∗*p* < 0.0001; *n.s.*, not significant). CPD, cysteine protease domain; MARTX, multifunctional autoprocessing repeats-in-toxin.

The autocleavage activity of CPD_MO6-24/O_ was much greater than that of CPD_BAA87_ (Fig. 1*C*). To elucidate the role of the β-flap in the regulation of the enzymatic function of CPD_BAA87_, we generated a chimeric CPD_BAA87_ (CPD_BAA87_/β-flap_MO6-24/O_) in which the β-flap is substituted with the β-flap of CPD_MO6-24/O_ (β-flap_MO6-24/O_). The chimeric CPD_BAA87_/β-flap_MO6-24/O_ showed very low autocleavage activity (Fig. 4*A*), suggesting that the parental β-flap region is necessary for autocleavage in conjunction with the extensional N-terminal region of CPD_BAA87_. Mutation of the unique sequence AWT to ALA in CPD_BAA87_/β-flap_MO6-24/O_ (CPD_BAA87/ALA_/β-flap_MO6-24/O_) to mimic CPD_MO6-24/O_ resulted in comparable autocleavage activity to that of CPD_MO6-24/O_ (Fig. 4*B*). Circular dichroism (CD) spectroscopy analysis revealed that there are no significant structural differences between CPD variants and WT CPD_BAA87_ (Fig. S4)

We further evaluated the roles of the extended N-terminal region containing the unique autocleavage site and the β-flap region in CPD_BAA87_ in MARTX toxin activation. Consistent with the previous results, CPD_BAA87_ was incapable of processing the effector domains within MARTX_BAA87_ even though it was autocleaved in the presence of InsP_6_ (Fig. 4*C*). The mutant CPD_BAA87/ALA_ showing similar autocleavage activity as CPD_MO6-24/O_ (Fig. 1*C*) also showed no effector processing activity (Fig. 4*C*). However, CPD_BAA87_/β-flap_MO6-24/O_ was processed between the Rho GTPase-inactivation domain (RID) and the alpha/beta hydrolase domain (ABH), although its autocleavage activity was reduced, as shown in Figure 4*A*, suggesting that this chimeric CPD forms an open conformation of the active site. The mutant CPD_BAA87/ALA_/β-flap_MO6-24/O_ showed similar processing activity as CPD_BAA87_/β-flap_MO6-24/O_ (Fig. 4*C*). Consistent with these results, the affinities of the chimeric proteins for InsP_6_ were lower than that of the WT (Fig. S5). These data suggest that the extended N-terminal region, including the distinct autocleavage site VLE and the β-flap in CPD_BAA87_, has evolved to have moderate autocleavage activity and reduced effector domain processing, which may significantly reduce the MARTX toxin-mediated virulence of the biotype 3 strain.

### The structural modulation of CPD_BAA87_ reduces virulence *in vivo*

**Figure 5:**
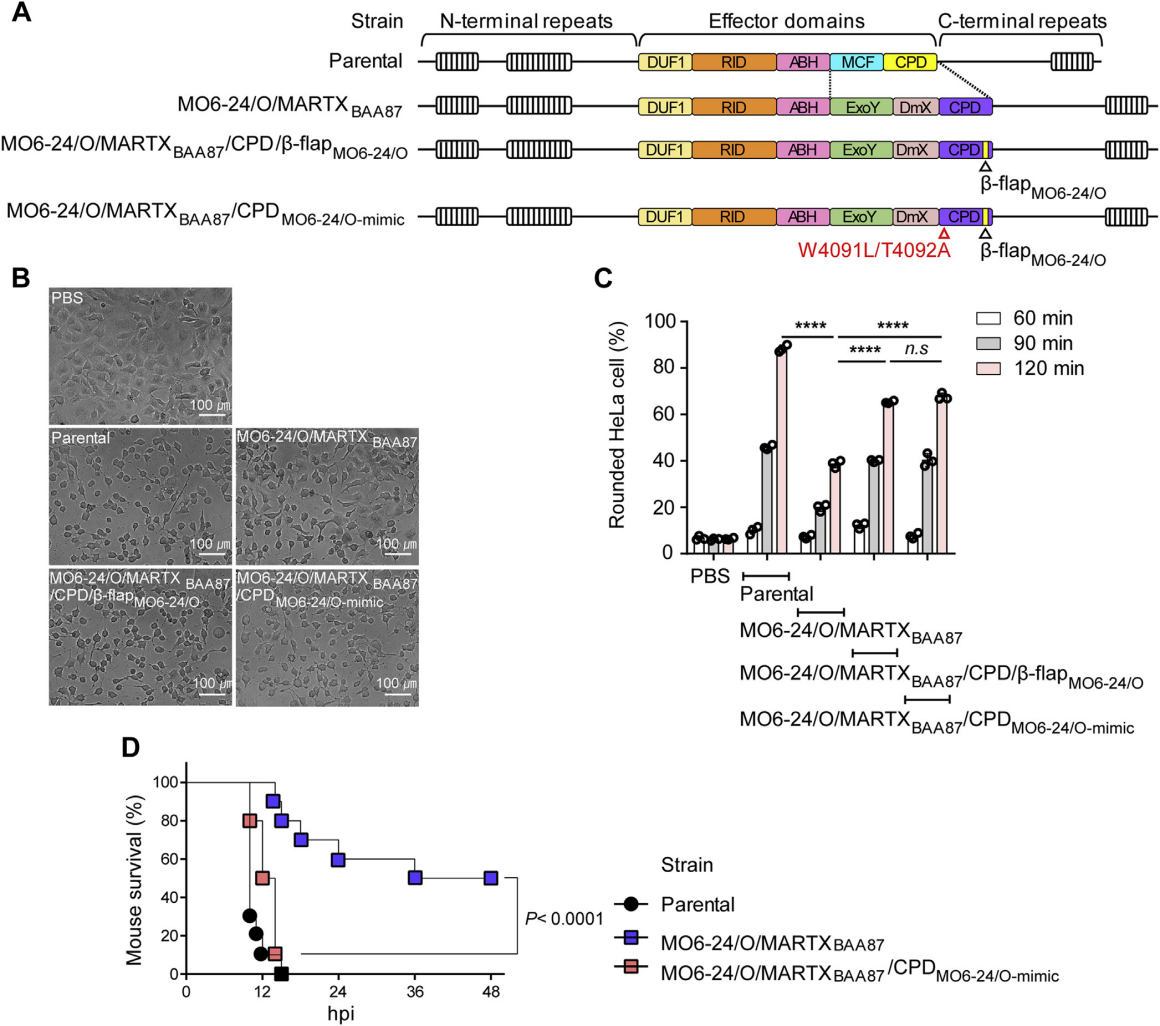
**The reduced virulence of the MARTX**_**BAA87**_**toxin is mediated by CPD**_**BAA87**_**.***A*, schematic representation of the *V. vulnificus* strains used for cell rounding assays and mouse survival experiments. Parental, *V. vulnificus* MO6-24/O strain; MO6-24/O/MARTX_BAA87_, *V. vulnificus* MO6-24/O expressing MARTX_BAA87_ (*i.e.*, in which the MCF and CPD of MARTX_MO6-24/O_ are substituted with the ExoY, DmX, and CPD of MARTX_BAA87_); MO6-24/O/MARTX_BAA87_/CPD/β-flap_MO6-24/O,_ an engineered strain in which the β-flap of CPD_BAA87_ is substituted with that of MO6-24/O; MO6-24/O/MARTX_BAA87_/CPD_MO6-24/O-mimic_, an engineered strain in which CPD_BAA87_ in MO6-24/O/MARTX_BAA87_ is substituted with CPD_MO6-24/O_. Mutated residues and the substituted regions in CPD_MO6-24/O-mimic_ are indicated. *B*, representative images of HeLa cells treated with PBS as a control or the indicated *V. vulnificus* strains at an multiplicity of infection of 0.1 for 120 min. *C*, quantification of rounded HeLa cells from images obtained after 60, 90, and 120 min of the treatments described in *B*. The proportion of rounded HeLa cells relative to the total number of HeLa cells was calculated. Data are shown as the mean ± standard deviation (SD) from three independent images (∗∗∗∗*p* < 0.0001). *D*, survival of mice challenged by subcutaneous injection of the indicated *V. vulnificus* strains (*n* = 10; pooled data from two independent experiments with five mice per group). Data from the MO6-24/O/MARTX_BAA87_ strain and the MO6-24/O/MARTX_BAA87_/CPD_MO6-24/O-mimic_ strain were compared by the log-rank test. CPD, cysteine protease domain; MARTX, multifunctional autoprocessing repeats-in-toxin.

Full activation of the MARTX toxin effector domains depends on internal proteases such as CPD and MCF that cleave the toxin, releasing the effector domains, which dysregulate host cell functions (16, 17). To assess whether the processing activity of CPD_BAA87_ directly influences the virulence of the MARTX toxin, we utilized a variant of the *V. vulnificus* MO6-24/O strain in which exotoxins including hemolysin, elastase, secretory protease, and secretory phospholipase A_2_ were deleted to evaluate MARTX toxin-specific effects (16). The MARTX toxin of the engineered *V. vulnificus* MO6-24/O strain (the parental strain) was substituted with MARTX_BAA87_ by exchanging the MCF and CPD_MO6-24/O_ with the ExoY-like adenylate cyclase domain (ExoY), DmX, and CPD_BAA87_ of MARTX_BAA87_ (MO6-24/O/MARTX_BAA87_ strain) (Fig. 5*A*). The CPD_BAA87_ of the MO6-24/O/MARTX_BAA87_ strain was further engineered into a CPD_MO6-24/O_-mimicking enzyme (MO6-24/O/MARTX_BAA87_/CPD_MO6-24/O-mimic_ strain) (Fig. 5*A*).

We first evaluated the virulence of the engineered strains in infected HeLa cells by measuring cell rounding, which is caused by inhibition of Rho GTPase *via* the effector RID within the MARTX toxin (22, 23). Most cells infected with the parental strain became rounded by 120 min, whereas cell rounding was significantly reduced in cells infected with the MO6-24/O/MARTX_BAA87_ strain (Fig. 5, *B* and *C*). The strain containing the CPD_MO6-24/O_-mimicking enzyme showed comparable virulence to the parental strain (Fig. 5, *B* and *C*). These results further suggest that the structural modulation of CPD_BAA87_ is linked to the significantly reduced virulence of the biotype 3 strain through inhibition of MARTX toxin processing.

We further assessed the significance of MARTX toxin processing by CPD_BAA87_ during the pathogenesis of *V. vulnificus in vivo*. Mice challenged with the MO6-24/O/MARTX_BAA87_ strain showed 50% mortality at 48 h postinfection (hpi), whereas the parental strain resulted in 100% mortality within 15 hpi (Fig. 5*D*). Strikingly, no mice challenged with the strain containing the CPD_MO6-24/O_-mimicking enzyme survived at 15 hpi, which is comparable with the mortality of the parental strain, further demonstrating that the structural modulation of CPD_BAA87_ is the determinant of the virulence moderation of *V. vulnificus* biotype 3 strains.

## Discussion

Opportunistic pathogens evolutionarily alter their virulence for adaptation to the environment and to facilitate infection of susceptible hosts (24). In this study, we found that the opportunistic pathogen *V. vulnificus* biotype 3, which caused an outbreak in humans associated with tilapia farming, may have evolved a form of an MARTX toxin with attenuated virulence. This may have allowed it to persist in the host for a longer time. MARTX toxins are expressed across multiple bacterial species and genera and are considered the primary virulence factors of *V. vulnificus* (13, 25). Once translocated into host cells, MARTX toxins undergo a proteolytic processing event *via* their internal CPD that releases functionally discrete effector domains into the host cell to dysregulate cellular substrates (16, 17, 18).

Genome-wide single nucleotide polymorphism genotyping revealed that the *V. vulnificus* biotype 3 genome is based on the core genome of a biotype 1 strain belonging to the clade B group (26). Evolutionary analysis of the *rtxA1* genes of various *V. vulnificus* strains suggested that the biotype 3 MARTX toxin may have been generated from the MARTX toxin of a biotype 1 clade B strain *via* a recombination event (10, 27). The biotype 3 MARTX toxin contains five effector domains, including domain of unknown function (DUF1), RID, ABH, ExoY, and DmX (a homolog of MCF), whereas a representative biotype 1 MARTX toxin consists of DUF1, RID, ABH, MCF, and Ras/Rap1-specific endopeptidase domain (RRSP). It has been proposed that modification of the effector domain content in the biotype 3 MARTX toxin results in altered toxin potency and contributes to the emergence of *V. vulnificus* strains with outbreak potential (10). That study demonstrated that restoring the biotype 3 toxin to the biotype 1 progenitor toxin by replacing the ExoY and DmX domains with MCF and RRSP significantly increased virulence in mice. However, that study replaced the effector domains ExoY and DmX as well as the CPD with MCF, RRSP, and CPD from biotype 1 strain LOS6966.

**Figure 6:**
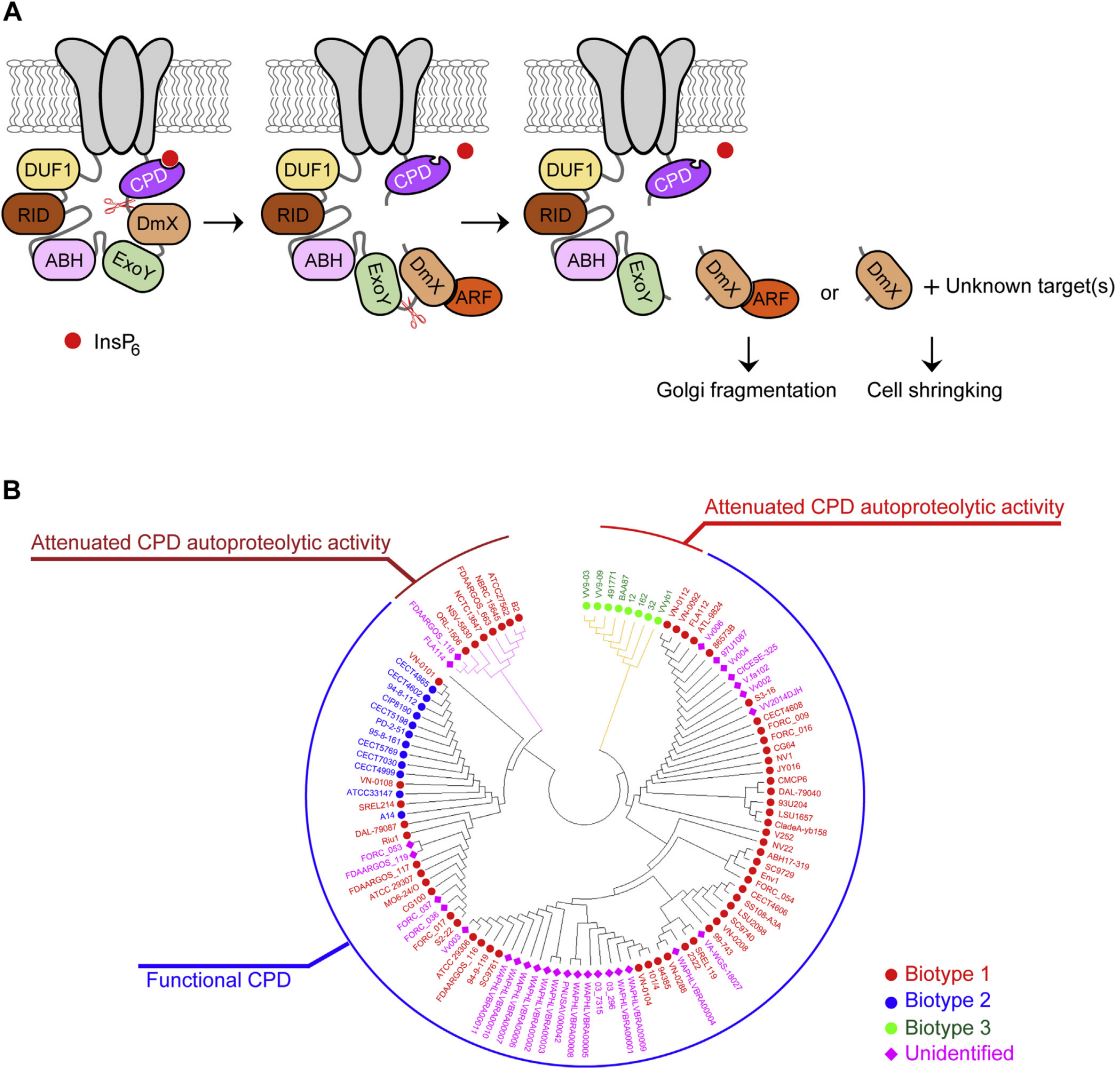
**The functionality of CPDs correlates with the evolutionary classification of *V. vulnificus* biotypes.***A*, proposed model for MARTX_BAA87_ processing. Upon entry into host cells, the CPD of MARTX_BAA87_ is allosterically activated by binding to InsP_6_ and autocleaved (*left panel*). DmX released from the autocleaved CPD is then allosterically activated *via* the interaction with ARF and autocleaved (*middle panel*). The released DmX complexed with ARF disrupts the Golgi structure, and DmX may induce cell shrinking *via* modification of an unknown target(s) (16, 28). *B*, phylogenetic tree of CPDs from different *V. vulnificus* strains. Amino acid sequence analysis revealed that CPDs can be classified into three groups based on their sequences and predicted functional discrepancies, shown as arcs outside of the tree. The biotypes of *V. vulnificus* strains are indicated with different symbols: biotype 1, *red circle*; biotype 2, *blue circle*; biotype 3, *green circle*; unidentified, *magenta diamond*. ARF, ADP-ribosylation factor; CPD, cysteine protease domain; DmX, domain X effector; DUF1, domain of unknown function; ExoY, ExoY-like adenylate cyclase domain; MARTX, multifunctional autoprocessing repeats-in-toxin; RID, Rho GTPase-inactivation domain.

Sequence alignment revealed that the CPD of the biotype 1 strain LOS6966 is a conventional CPD, enabling it to process associated effector domains (Fig. S6). We previously reported that the MCF homolog DmX in the biotype 3 MARTX toxin is autocleaved *via* allosteric activation by host ARF (16). Another study also showed that DmX is N terminally autocleaved by the interaction with ARF (28). These data, together with the present results, led us speculate that upon entry into host cells, the biotype 3 MARTX toxin undergoes autocleavage of the internal CPD by binding to InsP_6_ in the cytoplasm (Fig. 6*A*, left panel), and then, DmX is cleaved from the ExoY by activation of ARF (Fig. 6*A*, middle panel). Consequently, DmX is released from the toxin, but other effectors are not, because it becomes a nonfunctional protease after autocleavage of the CPD (Fig. 6*A*, right panel). This atypical MARTX toxin processing event may be related to the significantly attenuated virulence of the biotype 3 strain, rather than the modification of effector content. Substitution of the CPD with a conventional CPD would result in release of DUF1, RID, ABH, ExoY, and DmX from the toxin, significantly increasing the virulence of the pathogenic strain. It should be mentioned that a conventional CPD-like CPD_MO6-24/O_ does not cleave between DUF1 and RID (16). Note that the free forms of RID, ABH, ExoY, and DmX target and dysregulate Rho-family GTPases (22, 29), autophagy and endosome pathways (30), the cAMP signaling pathway (31), and the Golgi, as well as an unknown substrate(s) (16, 28), respectively.

Intriguingly, sequence analysis of CPDs from *V. vulnificus* MARTX toxins revealed that the functionality of the CPD correlates with the biotype. This indicates that most MARTX toxins of biotype 1 and 2 strains preserve conventionally functional CPDs for both self-cleavage and associated effector domain processing, whereas all biotype 3 strains retain attenuated CPD autoproteolytic activity (Fig. 6*B*).

Strong evidence suggests that climate change is influencing outbreaks and changing the epidemiology of *Vibrio* infections on a worldwide scale and will increase the spread of aquatic *Vibrio* pathogens, with detrimental effects on human and animal health (32, 33, 34). Although the *V. vulnificus* biotype 3 strain was originally isolated from outbreaks associated with tilapia farming in Israel (8), a biotype 3–associated case with primary septicemia was also reported in Japan (35), indicating that the habitat of the *V. vulnificus* biotype 3 has extended to East Asia and might spread worldwide in the future. Our study providing new insights into the mechanisms by which opportunistic bacteria make evolutionary trade-offs to increase their fitness in the environmental reservoir should expand our understanding of the regulation of virulence across a wide range of emerging infectious diseases.

## Experimental procedures

### Bacterial strains, plasmids, and culture media

The bacterial strains and plasmids used in this study are listed in Table S2. *Escherichia coli* and *V. vulnificus* strains were grown in Luria-Bertani medium at 37 °C and in Luria-Bertani supplemented with 2.0% (w/v) NaCl at 30 °C, respectively, with appropriate antibiotics.

### DNA cloning and protein purification

Genomic DNA of *V. vulnificus* strain BAA87 or MO6-24/O (16) was used as a template for PCR-based amplification of the DNA fragments encoding effector domains (residues 1959–4060 of MARTX_BAA87_ toxin and residues 1959–3586 of MARTX_MO6-24/O_ toxin) or CPD (residues 4056–4300 of MARTX_BAA87_ toxin and residues 3586–3796 of MARTX_MO6-24/O_ toxin) using the primers listed in Table S3. The resulting PCR products were treated with restriction enzymes corresponding to sites present in the primers and ligated into linearized pET21d, pProEX, or pPosKJ vectors for protein expression. To construct the pHis-parallel1 vector expressing chimeric CPD_BAA87_ (residues 4056–4300) in which the β-flap (residues 4265–4291 of CPD_BAA87_) was substituted with that of CPD_MO6-24/O_ (residues 3760–3786), DNA fragments encoding the flanking region of β-flap_BAA87_ (11,521–12,792 bp and 12,874–13,591 bp of *rtxA1* from *V. vulnificus* strain BAA87) and β-flap_MO6-24/O_ (11,278–11,358 bp of *rtxA1* from *V. vulnificus* strain MO6-24/O) were amplified, respectively, and simultaneously inserted into pHis-parallel1 using the *Nco*I/*Xho*I restriction sites *via* a one-step sequence- and ligation-independent cloning method (36). The DNA fragment encoding chimeric CPD_BAA87_ was amplified and ligated with *Nde*I/*Xho*I-digested pPosKJ to generate pPosKJ_CPD_BAA87_/β-flap_MO6-24/O_. In addition, pPosKJ_CPD_BAA87/ALA_ and pPosKJ_CPD_BAA87/ALA_/β-flap_MO6-24/O_ expressing CPD_BAA87/ALA_ or CPD_BAA87/ALA_/β-flap_MO6-24/O_, respectively, were produced by site-directed mutagenesis using pPosKJ_CPD_4056-4300_ or pPosKJ_CPD_BAA87_/β-flap_MO6-24/O_, respectively, as a template. Each plasmid was transformed into the *E. coli* NiCo21 (DE3) strain (New England Biolabs), and transformed cells were grown to an absorbance at 600 nm (*A*_600_) of 0.5 to 0.6. Expression of recombinant proteins was induced by adding 0.5 mM isopropyl-β-D-thiogalactopyranoside (LPS Solution). Transformed cells were further incubated at 18 °C for 18 h and harvested by centrifugation at 4000*g* for 5 min. The harvested cells were resuspended in buffer A [300 mM NaCl, 50 mM Tris-HCl (pH 7.5), 5% glycerol, and 1 mM DTT] supplemented with 10 mM imidazole and lysed with a high-pressure homogenizer (Nano DeBEE, B.E.E. International). The lysates were centrifuged at 28,000*g* at 4 °C for 1 h. The supernatants were loaded onto a column with nickel-nitrilotriacetic acid resin (Qiagen). The column was washed with 1 l of buffer A supplemented with 20 mM imidazole. Then, the hexa-histidine (His_6_)-tagged or His_6_-VHb-tagged recombinant proteins were eluted with buffer A supplemented with 250 mM imidazole. If necessary, the His_6_ tag or VHb tag was removed by treatment with recombinant tobacco etch virus protease during dialysis against buffer B [150 mM NaCl, 50 mM Tris-HCl (pH 7.5), 5% glycerol, and 1 mM DTT] at 4 °C for 10 h. Nonspecific or undigested proteins were eliminated by loading onto nickel-nitrilotriacetic acid resin and further purified by size-exclusion chromatography using a HiLoad 16/60 Superdex 75 column (GE Healthcare Life Science) preequilibrated with buffer B. The purified proteins were concentrated using Amicon Ultra-15 100 K or 10 K columns (EMD Millipore), flash-frozen in liquid nitrogen (LN_2_), and kept at −80 °C until use.

### Crystallization, X-ray diffraction, and structure determination

The DNA fragment encoding CPD (residues 4090–4300) was cloned into pHis-parallel1 using *Nco*I/*Xho*I restriction sites, and recombinant proteins were purified as described above. Specifically, the partially purified CPDs were incubated with a five-fold excess of InsP_6_ at 37 °C for 1 h in cleavage assay buffer [60 mM NaCl, 20 mM Tris-HCl (pH 7.5), and 250 mM sucrose] (17). The activated CPDs were further purified by size-exclusion chromatography using a HiLoad 16/60 Superdex 75 column (GE Healthcare Life Science). Finally, purified CPDs were concentrated to 21 mg/ml using an Amicon Ultra-15 10 K column (EMD Millipore) in buffer B, and after the addition of 2 mM InsP_6_, crystals were grown in 96-well MRC crystallization plates (Swissci) *via* the sitting-drop vapor-diffusion method. Crystals were produced at 20 °C after 3 days in reservoir solution containing 1.0 M potassium sodium tartrate and 0.1 M MES:NaOH (pH 6.0). For X-ray diffraction experiments, the CPD crystals were cryo-protected using the reservoir solution supplemented with 23% glycerol and stored in LN_2_ until diffraction. Then, X-ray diffraction data were collected at 2.2 Å resolution from the CPD crystals at beamline 5C at the Pohang Accelerator Laboratory. X-ray diffraction data were processed and scaled using the HKL2000 software package (37). The initial structure of CPD_BAA87_4090-4300_ was solved by molecular replacement in Phaser-MR from PHENIX using the *V. cholerae* CPD structure (PDB code, 3EEB) as a template model (17, 38). Model building was carried out with the COOT program, and refinement, including the translation-liberation-screw procedure, was implemented using phenix.refine from PHENIX (38, 39). The overall statistics of data collection and structure refinement are summarized in Table S1.

### *In vitro* processing assay and Edman sequencing

To investigate the autocleavage activity of CPDs (CPD_BAA87_, CPD_BAA87/ALA_, CPD_BAA87_/β-flap_MO6-24/O_, and CPD_BAA87/ALA_/β-flap_MO6-24/O_), the indicated CPDs (5 μM) were incubated at 37 °C for 1 h or the indicated durations in the absence or presence of InsP_6_ (5 μM) in cleavage assay buffer. The reaction was stopped by addition of SDS-PAGE sample buffer and boiling at 98 °C for 5 min. Proteins were loaded, separated on 15% polyacrylamide gels, and visualized by staining with Coomassie brilliant blue dye. The band intensities of cleaved and uncleaved CPDs from three independent experiments were quantified using the ImageJ program of the National Institutes of Health (40) and plotted using GraphPad Prism (version 6). For identification of the autocleavage site of CPD_BAA87_, N-terminally His_6_-tagged CPD_BAA87_ (residues 4090–4300) or His_6_-VHb-fused CPD_BAA87_ (residues 4056–4300) was incubated with InsP_6_ at 37 °C for 1 h in cleavage assay buffer. The cleaved CPD_BAA87_ was further purified by size-exclusion chromatography on a Superdex 75 10/300 GL column (GE Healthcare Life Science) preequilibrated with buffer B. Then, the cleaved CPD_BAA87_ was concentrated using an Amicon Ultra-15 10 K filter (EMD Millipore), and N-terminal amino acid sequences were analyzed using a PPSQ-51A protein sequencer (Shimadzu). In addition, following autocleavage, the N-terminal amino acid sequences of CPD_AWT/ALA_ and CPD_VLE/ALA_ were confirmed by Edman sequencing.

To analyze the effector domain-processing activity of the CPDs, the purified effector domains of MARTX _BAA87_ toxin (residues 1959–4060, 0.05 mg/ml) were incubated with the indicated CPDs (CPD_BAA87_, CPD_BAA87/ALA_, CPD_BAA87_/β-flap_MO6-24/O_, and CPD_BAA87/ALA_/β-flap_MO6-24/O_; 0.05 mg/ml) in the absence or presence of InsP_6_ (1 mM). The reactions were separated on 10% polyacrylamide gels and visualized by staining with Coomassie brilliant blue dye. Additionally, the proteins were transferred to Immobilon-P polyvinylidene fluoride membranes (0.45 μm pore size; EMD Millipore) before staining, and the N-terminal amino acid sequences of the processed effector domains were analyzed.

### Isothermal titration calorimetry analysis

To measure the binding affinity between CPD_BAA87_ and InsP_6_, isothermal titration calorimetry measurements were carried out using a VP-isothermal titration calorimetry Microcalorimeter (Microcal). Following the purification of enzymatically inactive CPD_C/S_ (residues 4056–4300; C4232S) and CPD_C/S_ (residues 4090–4300; C4232S) as described above, the proteins were dialyzed against buffer B for 12 h at 4 °C. Proteins (80 μM) were degassed for 20 min using a ThermoVac (Microcal), then placed in the reaction cell, and InsP_6_ (1 mM) dissolved in the same dialyzed buffer was inserted into the syringe for titration. The reaction heat data from InsP_6_ in the syringe into the buffer in the cell was subtracted from that of InsP_6_ in the syringe into the protein in the cell. All data were processed and fitted using the Origin program (version 7) supplied with the instrument. The chimeric proteins, CPD_BAA87_/β-flap_MO6-24/O_C/S(4056–4300) and CPD_BAA87_/β-flap_MO6-24/O_C/S(4090–4300), were prepared in the same manner as described above. Following dialysis, 0.75 mM InsP_6_ in a syringe was titrated into 32 μM of each chimeric protein in the reaction cell. The data were processed using the Origin program (version 7).

### Construction of *V. vulnificus* mutant strains

To evaluate the effects of MARTX toxin processing in cultured cells and mice, an engineered *V. vulnificus* MO6-24/O strain in which genes encoding other exotoxins [*vvhA* (hemolysin), *vvpE* (elastase), *plpA* (secretory phospholipase A2), and *vvpM* (secretory protease)] were deleted was utilized as a parental strain for the construction of *V. vulnificus* mutant strains (16). To construct plasmids expressing pDS_exoY/dmX/cpd, pDS_exoY/dmX/cpd_BAA87_/β-flap_MO6-24/O_, and pDS_exoY/dmX/cpd_BAA87/ALA_/β-flap_MO6-24/O_, two DNA fragments corresponding to the upstream region of ABH (573 bp-long) and downstream region of CPD (588 bp-long) of MARTX_MO6-24/O_ were cloned into *Sph*I/*Sac*I-digested pDS132 using the one-step sequence- and ligation-independent cloning method. ExoY/dmX/cpd, exoY/dmX/cpd_BAA87_/β-flap_MO6-24/O_, and exoY/dmX/cpd_BAA87/ALA_/β-flap_MO6-24/O_ were PCR-amplified and cloned between the two DNA fragments in pDS132. These constructs were then conjugally transferred into the parental strain to generate the MO6-24/O/MARTX_BAA87_, MO6-24/O/MARTX_BAA87_/CPD/β-flap_MO6-24/O_, and MO6-24/O/MARTX_BAA87_/CPD_MO6-24/O-mimic_ strains.

### Quantification of round HeLa cells and mouse survival experiments

To analyze CPD-mediated cytotoxicity by *V. vulnificus* variants, HeLa cells (5 × 10^5^/well) grown in 6-well plates (ThermoFisher Scientific) were treated with PBS (mock) or *V. vulnificus* (the parental strain, the MO6-24/O/MARTX_BAA87_ strain, MO6-24/O/MARTX_BAA87_/CPD/β-flap_MO6-24/O,_ or the MO6-24/O/MARTX_BAA87_/CPD_MO6-24/O-mimic_ strain) at an multiplicity of infection of 0.1. Images of three random fields from each well were obtained at the indicated time-points using the Floid Cell Imaging Station (ThermoFisher Scientific), and rounded HeLa cells were manually counted using the cell counter plug-in of the NIH ImageJ program. For mouse survival tests, 5-week-old female ICR (CrljOri:CD1) mice were purchased from Orientbio and adapted to the laboratory environment for 24 h. Parental, MO6-24/O/MARTX_BAA87_, or MO6-24/O/MARTX_BAA87_/CPD_MO6-24/O-mimic_ strains of *V. vulnificus* were grown to an *A*_600_ of 0.5 in Luria-Bertani supplemented with 2.0% (w/v) NaCl and harvested by centrifugation at 1000*g* for 3 min. The bacterial cells were washed once and diluted to 5 × 10^7^ colony forming units/ml using PBS. Subsequently, mice (*n* = 10 per each group) were subcutaneously administered 50 μl of the bacterial suspension on the dorsal side under light isoflurane anesthesia (10, 16) and were monitored every 2 h for 48 hpi. The Institutional Animal Care and Use committee of the Korea Research Institute of Bioscience and Biotechnology approved all mouse experiment protocols (approval no. KRIBB-ACE-18186).

### Analysis of CPDs and construction of the phylogenetic tree

Amino acid sequences corresponding to the CPDs of registered MARTX toxins from different *V. vulnificus* strains were downloaded from the National Center for Biotechnology Information database (https://www.ncbi.nlm.nih.gov/protein) and aligned using ClustalW. The sequences were analyzed by the maximum likelihood method, and the phylogenetic tree was constructed using Molecular Evolutionary Genetics Analysis X software (41). The CPDs were classified by comparing residues of the putative autocleavage sites and β-flap regions with those of CPD_BAA87_ and CPD_MO6-24/O_.

### Circular dichroism spectroscopy

CD spectroscopy was carried out to analyze and compare the structural conformations of CPD_BAA87_ and its variants. WT CPD_BAA87_ and its variants (0.1 mg/ml) in 150 mM NaCl, 50 mM Tris-HCl (pH 7.5), and 5% glycerol were subjected to CD spectroscopy at 20 °C using 1-mm path length quartz cuvettes and a Jasco J-815 CD spectrophotometer (Jasco). CD spectra were acquired over the wavelength range 200 to 260 nm and were converted into mean residue ellipticity (MRE, degree cm^2^ dmol^−1^).

## Data availability

The structure presented in this paper has been deposited in the Protein Data Bank, www.wwpdb.org (PDB ID code 7D5Y).

## Supporting information

This article contains supporting information (42, 43, 44, 45).

## Conflicts of interest

The authors declare that they have no conflicts of interest with the contents of this article.
