# Supplementary material for: Reduced virulence of the MARTX toxin increases the persistence of outbreak-associated Vibrio vulnificus in host reservoirs
Source: J Biol Chem. 2021 May 14;296:100777. doi: 10.1016/j.jbc.2021.100777 (PMC8191300; doi:10.1016/j.jbc.2021.100777)
Supplement: Supplemental Figures S1–S6 and Tables S1–S3 [file mmc1.docx]

**Supporting information**

**Table S1.**

Data collection and refinement statistics.

| Protein | | CPD_BAA87_ | | | | |
| --- | --- | --- | --- | --- | --- | --- |
| PDB ID | | 7D5Y | | |  |  |
| Space group | | *P4*_1_32 | | |  |  |
| X-ray source^a^ | | PAL-5C | | |  |  |
| Detector | | ADSC Q315 | | |  |  |
| Wavelength (Å) | | 0.9794 | | |  |  |
| Unit cell: *a*, *b*, *c* (Å) | | 125.31, 125.31, 125.31 | | |  |  |
| *α*, *β*, *γ* (º) | | 90.00, 90.00, 90.00 | | |  |  |
| Resolution range (Å)^b^ | | 50‒2.20  (2.24‒2.20) | | |  |  |
| *R*_merge_^c^ | | 11.70 (42.70) | | |  |  |
| *I*/σ*I* | | 103.69 (18.17) | | |  |  |
| Completeness (%) | | 99.98 (100.0) | | |  |  |
| Redundancy | | 41.60 (42.00) | | |  |  |
| Refinement | |  | | |  |  |
| Resolution range (Å) | | 34.75-2.20 | | |  |  |
| No. reflections | | 17586 (1720) | | |  |  |
| *R*_work_^d^ (%) / *R*_free_ (%) | | 18.50 (21.51) / 21.70 (25.77) | | |  |  |
| No. atoms / residues | | 1732 / 210 | | |  |  |
| Protein | | 1620 | | |  |  |
| Ligand (glycerol) | | 12 | | |  |  |
| Water | | 100 | | |  |  |
| B-factors (Å^2^) | | 49.60 | | |  |  |
| Protein | | 49.20 | | |  |  |
| Ligand (glycerol) | | 79.40 | | |  |  |
| Water | | 52.00 | | |  |  |
| Model statistics | |  | | |  |  |
| rmsd bond length (Å) | | 0.003 | | |  |  |
| rmsd bond angles (°) | | 0.70 | | |  |  |
| Ramachandran plot (%)  favored/allowed/disallowed | | 97.00/3.00/0 | | |  |  |
|  | ^a^ Beamline 5C and 7A at Pohang Acceleratory Laboratory (PAL) in South Korea.  ^b^Values in parentheses are for the highest-resolution shell.  ^c^*R*_merge_ =∑*_h_* ∑*_i_* │I(*h*)*_i_*−‹I(*h*)›│/ ∑*_h_* ∑*_i_*I(*h*)*_i_*, where I(*h*) is the intensity of reflection of *h*, ∑*_h_* is the sum over all reflections and ∑*_i_* is the sum over *i* measurements of reflection *h*.  ^d^ *R*_work_ = Σ*_hkl_* \|\|F*_o_*\|-\|F*_c_*\|\|/Σ*_hkl_*\|F*_o_*\|; 5% of the reflections were excluded for the *R*_free_ calculation. | | |  |  | |

**Table S2**

Bacterial strains and plasmids used in this study.

| **Strain/plasmid** | **Relevant genotype/phenotype/characteristics** | **Source** |
| --- | --- | --- |
| ***Escherichia coli*** | | |
| DH5α | *F^–^ Φ80lacZΔM15 Δ(lacZYA^–^argF) U169 recA1 endA1 hsdR17 (r_K_^–^, m_K_^+^) phoA supE44 λ^–^ thi-1 gyrA96 relA1* | Invitrogen |
| S17-1 λ *pir* | λ *pir* lysogen; *thi pro hsdR hsdM^+^ recA* RP4-2 Tc::Mu-Km::Tn7; Tp^R^ Sm^R^;  host for π-requiring plasmids; conjugal donor | (42) |
| NiCo21 (DE3) | *can::CBD fhuA2 [lon] ompT gal (λ DE3) [dcm] arnA::CBD slyD::CBD glmS6Ala ∆hsdS λ DE3 = λ sBamHIo ∆EcoRI-B int::(lacI::PlacUV5::T7 gene1) i21 ∆nin5* | NEB |
| ***Vibrio vulnificus*** | | |
| MO6-24/O | Wild type; clinical isolate; virulent; Biotype 1 | Laboratory collection |
| BAA87 (CDC 9530-96) | Wild type; clinical isolate; virulent; Biotype 3 | American Type Culture Collection |
| Parental | *V. vulnificus* MO6-24/O with *ΔvvhA ΔvvpE Δplp ΔvvpM* | (16) |
| MO6-24/O/MARTX_BAA87_ | Parental with *BAA87-rtxA1* (effector domains and CPD of MARTX_BAA87_ toxin) | This study |
| MO6-24/O/MARTX_BAA87_ /CPD/ β-flap_MO6-24/O_ | Parental with *BAA87-rtxA1::β-flap_MO6-24/O_-cpd_BAA87_* | This study |
| MO6-24/O/MARTX_BAA87_ /CPD_MO6-24/O-mimic_ | Parental with *BAA87-rtxA1::β-flap_MO6-24/O_-cpd_BAA87_W4091L/T4092A* | This study |
| **Plasmids** | | |
| pPosKJ | Amp^R^, a derivative of pKW32 with a bacterial hemoglobin (VHb) tag and a TEV cleavage site | (43) |
| pPosKJ_CPD_4056-4300_ | Amp^R^, wild type CPD (4056-4300, NdeI/XhoI) from *V. vulnificus* BAA87 MARTX in pPosKJ | This study |
| pPosKJ_CPD_ALA_ | Amp^R^, CPD (4056-4300, W4091L, T4092A; NdeI/XhoI) from *V. vulnificus* BAA87 MARTX in pPosKJ | This study |
| pPosKJ_CPD_BAA87_/β-flap_MO6-24/O_ | Amp^R^, CPD (4056-4300, substituted residues 4265-4291 with 3760-3786 of MO6-24/O MARTX; NdeI/XhoI) from *V. vulnificus* BAA87 MARTX in pPosKJ | This study |
| pPosKJ_CPD_BAA87/ALA_/β-flap_MO6-24/O_ | Amp^R^, CPD (4056-4300, W4091L, T4092A, substituted residues 4265-4291 with 3760-3786 of MO6-24/O MARTX; NdeI/XhoI) from *V. vulnificus* BAA87 MARTX in pPosKJ | This study |
| pPosKJ_CPD_VLE/ALA_ | Amp^R^, CPD (4056-4300, V4066A, E4068A; NdeI/XhoI) from *V. vulnificus* BAA87 MARTX in pPosKJ | This study |
| pET21d | Amp^R^, bacterial cell vector expressing His_6_ tag in the C-terminus of target protein | Novagen |
| pET21d_DUF1-RID-ABH-ExoY-DmX | Amp^R^, wild type DUF1-RID-ABH-ExoY-DmX (1959‒4060; NcoI/XhoI) from *V. vulnificus* BAA87 MARTX in pET21d | (16) |
| pHis-Parallel1 (pHisP) | Amp^R^, a derivative of pFastBac-HTa (NdeI) with a polylinker of pET22B, AF097413 | (44) |
| pHisP_CPD_4090-4300_ | Amp^R^, wild type CPD (4090-4300, NcoI/XhoI) from *V. vulnificus* BAA87 MARTX in pHis-Parallel1 | This study |
| pHisP_CPD_4090-4300_C/S | Amp^R^, CPD (4090-4300, C4232S; NcoI/XhoI) from *V. vulnificus* BAA87 MARTX in pHis-Parallel1 | This study |
| pHisP_CPD_4056-4300_C/S | Amp^R^, CPD (4056-4300, C4232S; NcoI/XhoI) from *V. vulnificus* BAA87 MARTX in pHis-Parallel1 | This study |
| pDS132 | Cm^R^, *oriR6K*, *sacB*, *oriT*, RP4 | (45) |
| pDS_flanking_CPD | Cm^R^, flanking region of CPD without CPD (3013-3212, 3797-3996; SphI/SacI) from *V. vulnificus* MO6-24/O MARTX in pDS132 | This study |
| pDS_exoY/dmX/cpd | Cm^R^, ExoY-DmX-CPD_WT_ (3213-4300; SphI/SacI) from *V. vulnificus* BAA87 MARTX in pDS_flanking_CPD | This study |
| pDS_exoY/dmX/cpd_BAA87/ALA_/β-flap_MO6-24/O_ | Cm^R^, ExoY-DmX-CPD_active_ (3213-4300, W4091L, T4092A, substituted residues 4265-4291 with 3760-3786 of MO6-24/O MARTX; SphI/SacI) in pDS_flanking_CPD | This study |
| pDS_exoY/dmX/cpd_BAA87_/β-flap_MO6-24/O_ | Cm^R^, ExoY-DmX-CPD_active_ (3213-4300, substituted residues 4265-4291 with 3760-3786 of MO6-24/O MARTX; SphI/SacI) in pDS_flanking_CPD | This study |
| Amp^R^, ampicillin-resistant; Cm^R^, chloramphenicol-resistant | | |

**Table S3**

Oligonucleotides used for cloning and site-directed mutagenesis in this study.

| **Oligonucleotide** | **Sequence (5' to 3')** | **Use** |
| --- | --- | --- |
| **pPosKJ vector** | | |
| CPD_4056-For | GGAATTCCATATGCAGAGTCATGCTGAAAGTG | Cloning |
| CPD_4300-Rev | CCGCTCGAGTTGACCATTCCAACTTAGCAAAAC | Cloning |
| CPD_C4232S_-For | CATATCAGTATTGTTGGTAGTTCTTTGGTGAGTGACG | Mutagenesis |
| CPD_C4232S_-Rev | CGTCACTCACCAAAGAACTACCAACAATACTGATATG | Mutagenesis |
| CPD_W4091L/T4092A_-For | CCTGGTCAGAAAGATGCGTTGGCTGGAGCGACTAGTAAGGC | Mutagenesis |
| CPD_W4091L/T4092A_-Rev | GCCTTACTAGTCGCTCCAGCCAACGCATCTTTCTGACCAGG | Mutagenesis |
| CPD_V4285D_-For | GATGGTAAGGATTCTTGGGATAACAAGGCTATAAACAGC | Mutagenesis |
| CPD_V4285D_-Rev | GCTGTTTATAGCCTTGTTATCCCAAGAATCCTTACCATC | Mutagenesis |
| CPD_L4276D_-For | TTACATCAATGAAATGGGACGAAAAGATTATTTTGATGGTAAGGATTCTTGG | Mutagenesis |
| CPD_L4276D_-Rev | CCAAGAATCCTTACCATCAAAATAATCTTTTCGTCCCATTTCATTGATGTAA | Mutagenesis |
| CPD_F4278E_-For | AAGTTTACATCAATGAAATGGGACGAAAACTTTATGAGGATGGTAAGGATTCTTGGG | Mutagenesis |
| CPD_F4278E_-Rev | CCCAAGAATCCTTACCATCCTCATAAAGTTTTCGTCCCATTTCATTGATGTAAACTT | Mutagenesis |
| CPD_V4066A/E4068A_-For | GCGACGATTACCGCGTTGGCGGTGAGTGGGCAA | Mutagenesis |
| CPD_V4066A/E4068A_-Rev | TTGCCCACTCACCGCCAACGCGGTAATCGTCGC | Mutagenesis |
| **pET21d vector** | | |
| DUF1-RID-ABH-ExoY-DmX_BAA87_-For | CATGCCATGGGTGAAGCTTCGCATGACTCTG | Cloning |
| DUF1-RID-ABH-ExoY-DmX_BAA87_-Rev | CCGCTCGAGTTCAGCATGACTCTGAATCGACAAC | Cloning |
| **pHis-Parallel-1 vector** | | |
| CPD_4090-For | CATGCCATGGCGTGGACTGGAGCGACTAGTAAG | Cloning |
| CPD_upstream_NcoI_SLIC-For | TTTCAGGGCGCCATGGGAGTATAGCGGTAAG | Cloning |
| CPD_upstream_XhoI_SLIC-Rev | GTGGTGGTGCTCGAGGCGAGTCCAGTTCAAC | Cloning |
| CPD_β-flap_upstream_SLIC-Rev | CTACGGCCAGTTCAGAACTGCGAACAGAAAC | Cloning |
| CPD_β-flap_downstream_SLIC-For | CAAAAAGCCGAAAACAACAAAGTTTTGCTAAGTTGG | Cloning |
| CPD_β-flap_MO6-24/O__SLIC-For | GTTTCTGTTCGCAGTTCTGAACTGGCCGTAG | Cloning |
| CPD_β-flap_MO6-24/O__SLIC-Rev | CAACTTAGCAAAACTTTGTTGTTTTCGGCTTTTTG | Cloning |
| **pDS132 vector** | | |
| MO6_CPD_upstream_XbaI_SLIC_F | GTTAAAAAGGATCGATCCTCTAGAGCAATCCAAC | Cloning |
| MO6_CPD_downstream_SacI_SLIC_R | CAATTTGTGGAATTCCCGGGAGTGTCCATTGCTG | Cloning |
| MO6_CPD_deletion_For | GATACGAAAGAAGCATTGGGTGAAGTTGTTGCC | Cloning |
| MO6_CPD_deletion_Rev | GGCAACAACTTCACCCAATGCTTCTTTCGTATC | Cloning |
| Hybrid_upstream_SphI_SLIC-For | GATCCCAAGCTTCTTCTAGAGGTACCGGCGAGCGAGATTCGCAACCAC | Cloning |
| Hybrid_downstream_SacI_SLIC-Rev | CAATTTGTGGAATTCCCGGGAGCAATGTCCATTGCTGCTCTTGAGCC | Cloning |
| Hybrid_ABH-ExoY_SLIC_Rev | GTCGCACGAATATCTTTCACTTCTACCGCTGCTTGCTCTG | Cloning |
| Hybrid_ExoY-DmX-CPD_SLIC_For | CAGAGCAAGCAGCGGTAGAAGTGAAAGATATTCGTGCGAC | Cloning |
| Hybrid_ExoY-DmX-CPD_SLIC_Rev | CATCCTTGGCAACAACTTCTTGACCATTCCAACTTAGC | Cloning |
| Hybrid_CPD-Cterm_SLIC-For | GCTAAGTTGGAATGGTCAAGAAGTTGTTGCCAAGGATG | Cloning |
| **pProEX vector** | | |
| CPD_MO6-24/O__For | CATGCCATGGCATTGGCTGGTGGAAAGATAC | Cloning |
| CPD_MO6-24/O__Rev | ATAAGAATGCGGCCGCTCAACCTTGCTCGTCCCAGCT | Cloning |


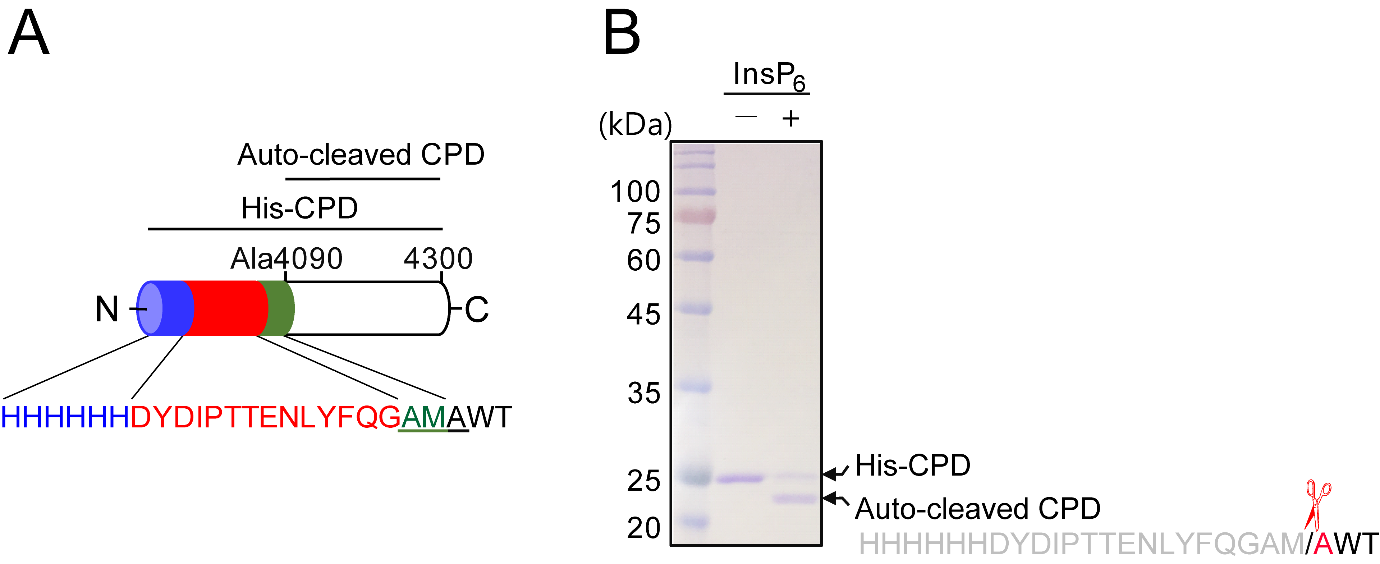


**Figure S1. Expression of crystallizable auto-cleaved CPD_BAA87_.** *A*, Schematic representation of the expression plasmid. When incubated with InsP_6_, the CPD is auto-cleaved at the AMA site, generating CPD starting from Ala4090. After incubation with InsP6, the auto-cleaved CPD was purified further for protein crystallization. The His tag, TEV protease recognition sequence, and cloning site sequence are shown in blue, red, and green, respectively. *B*, Auto-cleaved CPD_BAA87_ on SDS-PAGE. Auto-cleaved CPD starting from Ala4090 (red) was analyzed by Edman sequencing.


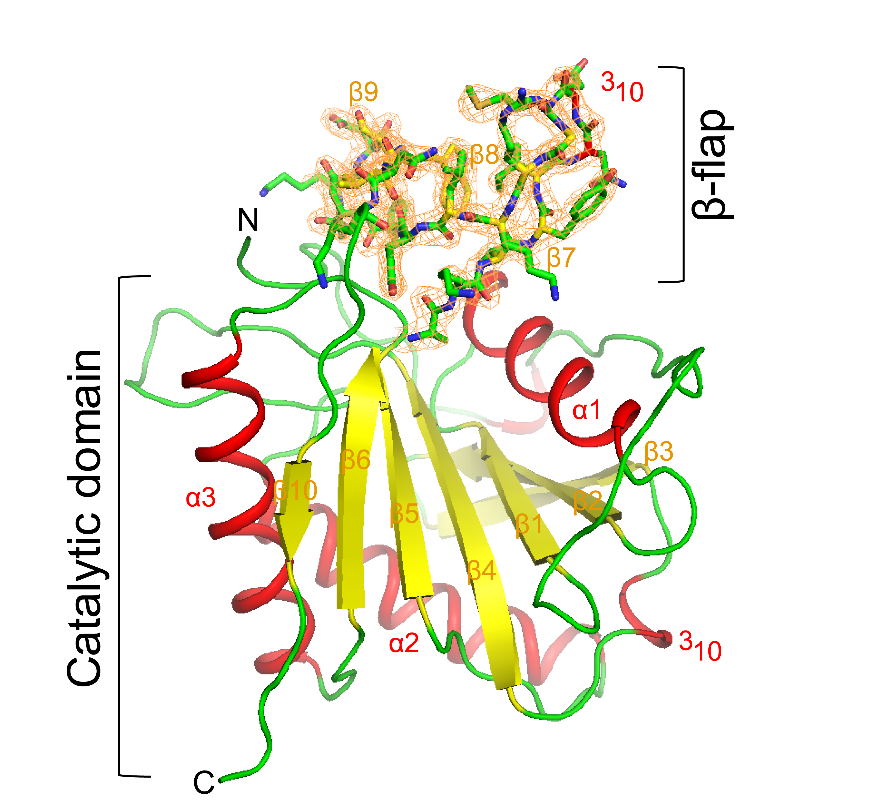


**Figure S2. The image of the 2Fo-Fc electron density map showing the β-flap region of CPD_BAA87_.** The electron density maps contoured at 2.0 σ are shown in orange for the β-flap region.


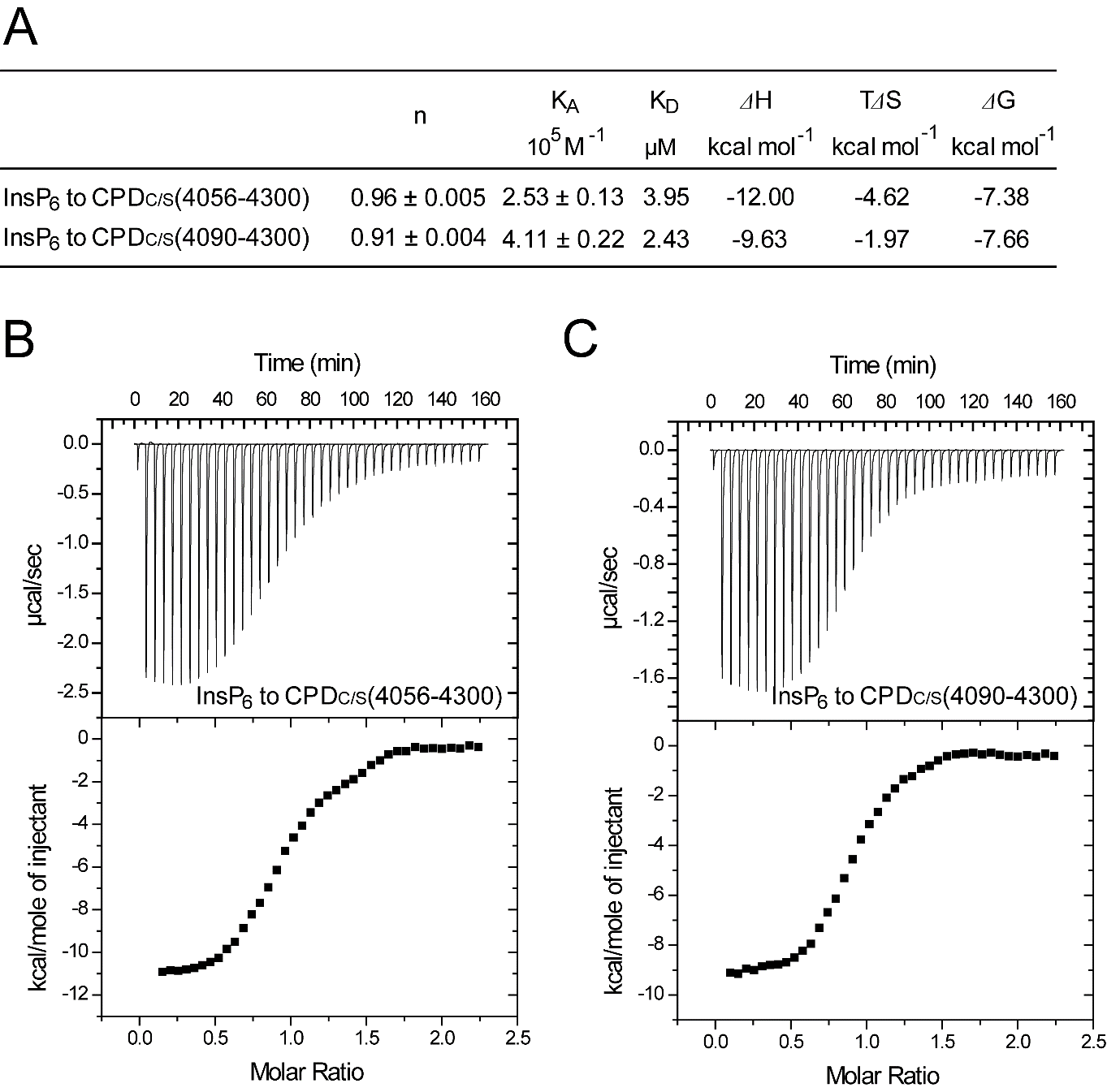


**Figure S3. ITC analysis of the binding affinity of CPD_BAA87_ and InsP_6_.** *A*, Binding parameters showing similar binding affinities of CPD_C/S_ (4056–4300) and CPD_C/S_ (4090–4300) with InsP_6_. *B* and *C*, Raw ITC data showing interactions between CPD_C/S_ (4056–4300) (B) or CPD_C/S_ (4090–4300) (C) and InsP_6_.


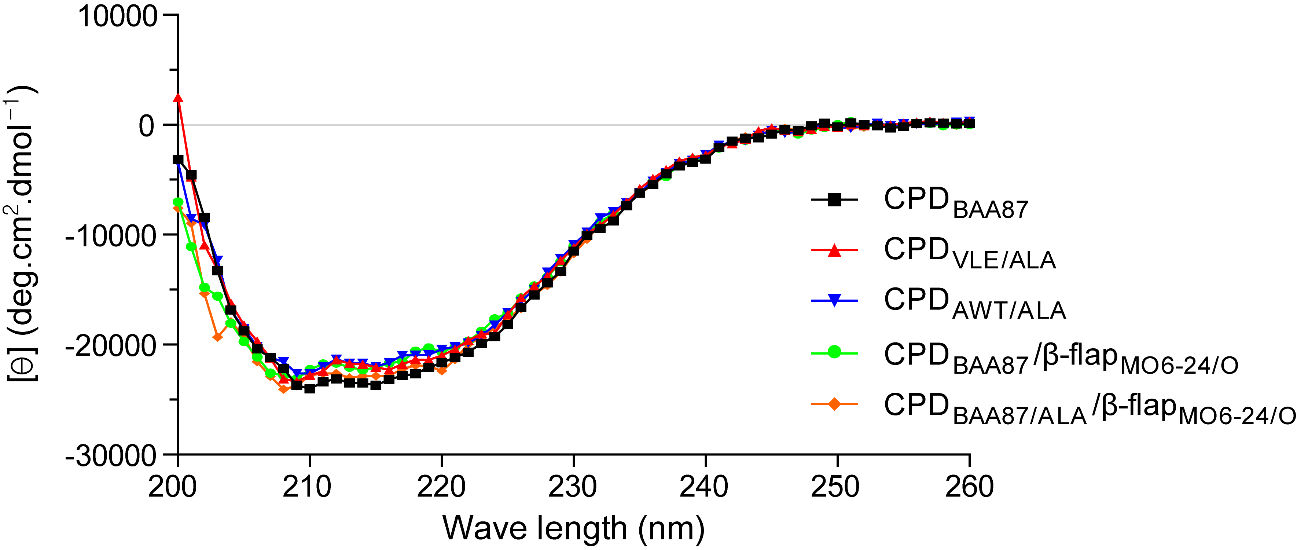


**Figure S4. Circular dichroism analysis of CPD_BAA87_ and its variants.** Typical far-UV spectra of CPD_BAA87_ and its variants.

**
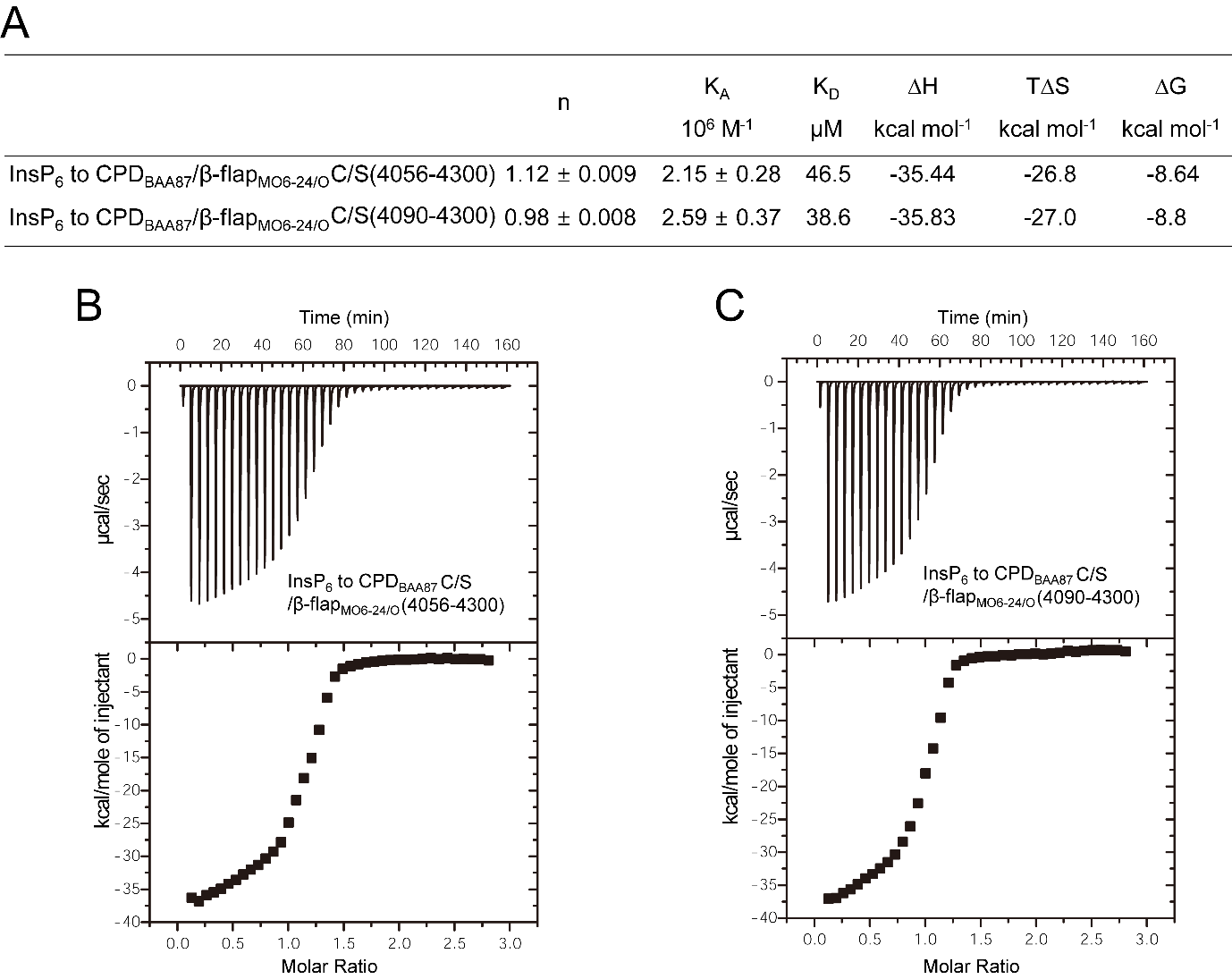
**

**Figure S5. ITC analysis of the binding affinity of the chimeric proteins (CPD_BAA87_/β-flap_MO6-24/O_C/S(4056-4300) and CPD_BAA87_/β-flap_MO6-24/O_C/S(4090-4300), respectively) to InsP_6_.** *A*, Binding parameters of CPD_BAA87_/β-flap_MO6-24/O_C/S(4056-4300) and CPD_BAA87_/β-flap_MO6-24/O_C/S(4090-4300) to InsP_6_. *B* and *C*, Raw ITC data showing interactions between CPD_BAA87_/β-flap_MO6-24/O_C/S(4056-4300) (B) or CPD_BAA87_/β-flap_MO6-24/O_C/S(4090-4300) (C) and InsP_6_.


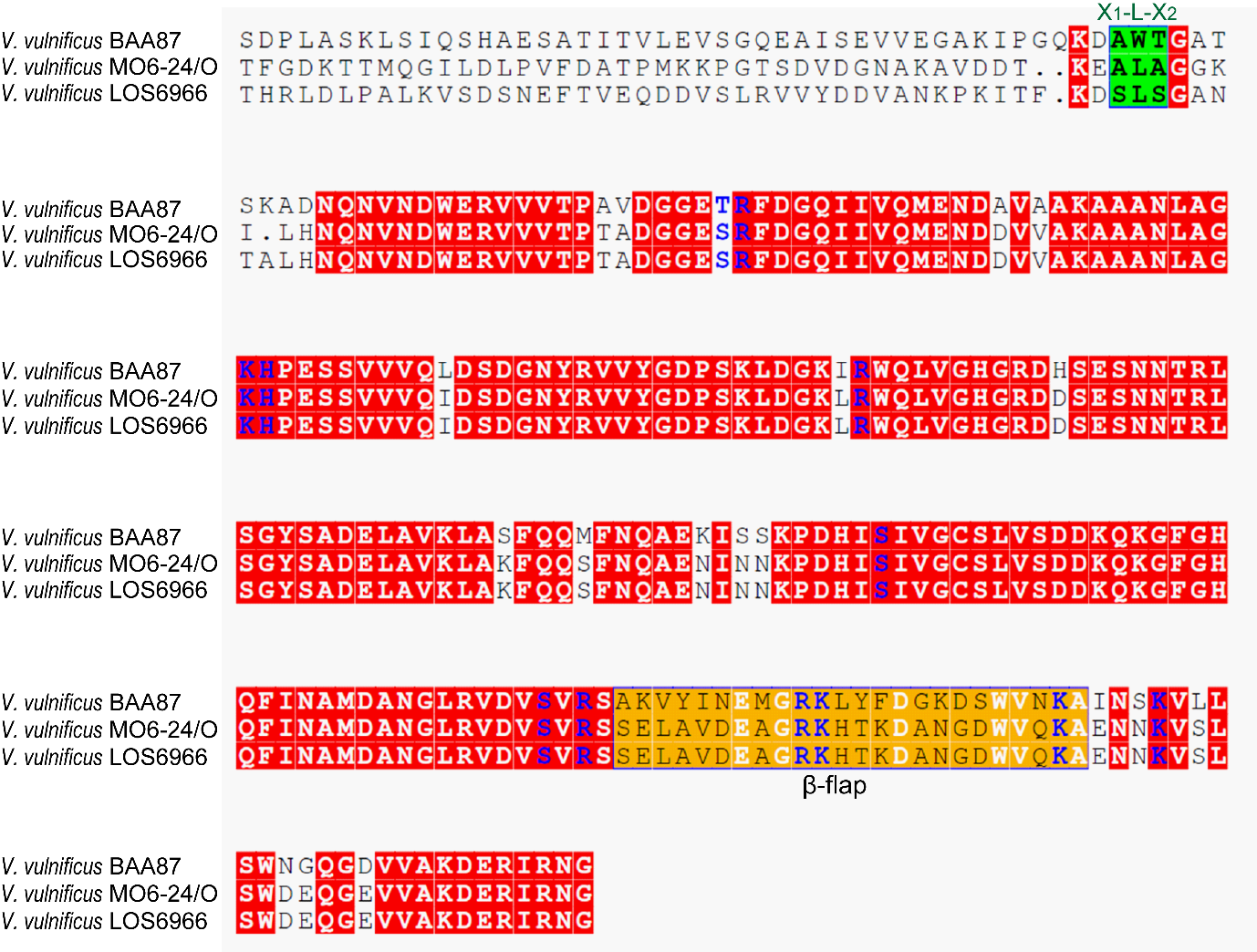


**Figure S6. The CPD of the biotype 1 strain LOS6966 is a conventional cysteine protease capable of processing associated effector domains.** Sequence alignment of CPDs from *V. vulnificus* strains. Strictly conserved residues, the β-flap region, and the X_1_-L-X_2_ motif are boxed in red, dark yellow, and green, respectively. Multiple sequence alignment was carried out using MultiAlin, and the figure was generated with ESPript.
